# Anoikis regulator GLI2 promotes NC cell immunity escape by TGF-β-mediated non-classic hedgehog signaling in colorectal cancer: based on artificial intelligence and big data analysis

**DOI:** 10.18632/aging.205283

**Published:** 2023-12-29

**Authors:** Zhang Shanshan, Ding Fanfei, Sun Xuan, Lu Huina, Zhang Ye, Li Jiayu, Zhao Shuo, Pan Xue, Pu Yingye, Jin Chengjun, Pan Hang, Li Li

**Affiliations:** 1 Laboratory Department of Changhai Hospital, First Affiliated Hospital of Naval Military Medical University, Shanghai, China; 2 Clinical Laboratory of PLA Naval Medical Center, Shanghai, China; 3 Medical Imaging Department of Changhai Hospital, First Affiliated Hospital of Naval Military Medical University, Shanghai, China

**Keywords:** anoikis, tumor immunity, drug tolerance, NK cell, machine learning

## Abstract

Background: Anoikis is a speed-limited procedure to inhibit tumor metastasis during epithelial-mesenchymal transition (EMT). Previous studies have explored anoikis-related genes (ARG) in predicting prognosis and distinguishing tumoral immunity in many types of cancer. However, the role of ARGs in regulating NK cell exhaustion (NKE) and in predicting chemotherapy sensitivity is not clear. Therefore, it is necessary to work on it.

Methods: Gene expression profiles and clinical features are collected from TCGA and GEO, and data analysis is performed in R4.2.0.

Results: The ARGs-based no-supervised learning algorithm identifies three ARG subgroups, amongst which the prognosis is different. WCGNA and Artificial intelligence (AI) are applied to construct an NKE-related drug sensitivity stratification and prognosis identification model in digestive system cancer. Pathways association analysis screens out GLI2 is a key gene in regulating NKE by non-classic Hedgehog signaling (GLI2/TGF-β/IL6). *In vitro* experiments show that down-regulation of GLI2 enhances the CAPE-mediated cell toxicity and accompanies with down-regulation of PD-L1, tumor-derive IL6, and snial1 whereas the expression of cleaved caspas3, cleaved caspase4, cleaved PARP, and E-cadherin are up-regulated in colorectal cancer. Co-culture experiments show that GLI2- decreased colorectal tumor cells lead to down-regulation of TIM-3 and PD1 in NK cells, which are restored by TGF-bate active protein powder. Besides, the Elisa assay shows that GLI2-decreased colorectal tumor cells lead to up-regulation of IFN-gamma in NK cells.

## INTRODUCTION

The overall incidence of Colorectal Cancer (CRC) ranks third in the world and is the second largest cause of cancer-related death [1]. In China, the proportion of advanced colorectal cancer in the first diagnosis is as high as 80%, of which more than 80% are difficult to accept radical surgery. Chemotherapy and immunotherapy provide possibilities for inhibiting CRC progression and R0-resection. However, due to drug resistance (DR), combination chemotherapy is still difficult to improve the 5-year survival rate of advanced CRC (about 12%), among which about 54% of patients have recurrence [2, 3]. Therefore, identification of CRC-sensitive drugs and propelling personalized treatment strategies are important to improving the efficacy of CRC.

The developing evidence shows that gene-based prognosis assessment systems have the potential abilities to predict the clinical outcome and give advice in making drug therapy regimens, such as ferroptosis-related genes [4, 5], cuproptosis-related genes [6, 7] or pyroptosis-related genes [8], et al. Anoikis is a speed-limited procedure for cancer metastasis and invasion [9], related genes of which are also reported to be used in constructing prognosis prediction models in colorectal cancer, and all of the related studies focus on prognosis assessment and immunotherapy response prediction [10, 11]. Therefore, it is necessary to explore the role of anoikis-related genes in assessing drug sensitivity in colorectal.

Artificial intelligence (AI) is already applied in disease diagnosis, such as Imaging and ECG. In this study, non-supervised and supervised AI algorithms are applied to construct a drug response assessment tool based on an anoikis-related gene (ARG) in colorectal cancer. Besides, a pivotal ARG is screened out, and is verified as a regulator of drug sensitivity and tumor immunity escape.

## MATERIALS AND METHODS

### Reagent

The colorectal cancer cell line (Lovo) and gastric cancer cell line (AGS) are purchased from the Cell Bank of the Chinese Academy of Sciences. The alive and dead staining kit is purchased from YEASEN (Shanghai, China). Capecitabine is purchased from CSNpharm. Antibodies against GLI2 (DF7541), RhoD (DF4439), GAPDH (AF7021), PD-L1 (BF8035), TGF-b (AF1027), snail1 (AF6032), E-cadherin (BF0219), IL6 (DF6087), cleaved-caspase3 (AF7022), cleaved-caspase4 (AF5373), cleaved PARP (AF7023) and vimentin (AF7013) are purchased from Affinity. RPMI-1604, MEM, OPTI-MEM, and FBS are purchased from Gibco. Penicillin and streptomycin are purchased from Beyotime Biotechnology (China). CCK-8 kit is purchased from Beyotime Biotechnology. RNA transfection reagent is purchased from Polyplus. BCA kit is purchased from Beyotime Biotechnology.

### Biological experiments

#### Cell culture

Lovo and AGS are cultured in RPMI-1640 with 10% FBS 1% penicillin and 1% streptomycin at 37°C and 5% humidity. NK-92 is cultured in MEM with 15% FBS, 1% penicillin, 1% streptomycin, 200 U/ml IL2, and 0.02 mM Folic Acid at 37°C and 5% humidity.

#### Alive and dead staining

According to protocol, cells are washed with PBS 3 times, followed by being washed with a 1-fold staining buffer for 5 mins. Then dilute working dye: 5 ml 1-fold buffer with 5 µl PI and 10 µl Calcein-AM. Adding working dye for another 30 mins culture. Next, wash the cell with PBS three times. Detect red and green fluorescence with a microscope. The positive area and the strength of staining are assessed by Image J.

#### Small interfere RNA transfection

40,000 cells are transplanted into a 6-well plate 24 h beforehand, followed by being cultured with OPTI-MEM for at least 2 h. Transfection system: 200 µl transfection buffer with 6 µl siRNA (20 uM) and 6 µl transfection reagent. Mix and stand for 15 minutes. Add it into cells. After 48 h, cells are harvested for western blot assay.

#### Western blot assay

Simply, cells are harvested by cell brush, and washed by precooling PBS. After centrifugation, cell precipitation is cleaved by RIPA for 30 minutes in ice water. 12000 g centrifugation to collect the supernatant. BCA kit is used to adjust total protein concentration as 1 ug/ul, for further electrophoresis and immunoblotting.

#### Cell viability detection cells

Are treated with different treatments in a 96-well plate and then cultured with CCK-8 dye for 4 h. Cell viability is calculated as follows:


$$\text{cell viability }(\%)=\frac{\text{value (experimental group}-\text{blank group)}}{\text{value (control group}-\text{blank group)}}$$

### Biological informatics analysis

#### Data collection

Gene expression array and clinical data are collected from The Cancer Genome Atlas (TCGA), Gene Expression Omnibus (GEO). UALCAN (The University Alabama at Birmingham Cancer Data Analysis Portal) is used to collect prognosis data. Gene lists of anoikis are collected from GeneCard (https://www.genecards.org). GSE39582 (*n* = 578) cohort is used for independent verification.

#### Multiple-genes-based risk model

Simply, Least Absolute Shrinkage and Selection Operator (LASSO) is used to screen out prognosis-related anoikis-related genes (ARGs). Multivariate Cox regression is used to construct a multiple genes risk model. The receptor operating curve (ROC) is performed to assess the prediction efficiency of the model.

#### Non-supervised machine learning

ARGs are put into consensus cluster with package *ConsensusClusterPlus* in R4.2.0.

#### Supervised artificial intelligence

Result of consensus-cluster-mediated pan-cancer grouping is as the baseline for five types of artificial intelligence algorithms, which include random forest (*randomForest*), Extreme Gradient Boosting (*xgboost*), Support Vector Machine (*e1071*), multi-logistic (*nnet*), and deep learning (*h2o*). During the analysis process, 75% of the TCGA cohort is put into the training cohort, and the last 30% is put into the test cohort.

#### Immune cell infiltration

Is predicted by CIBERSORT, *p* < 0.05 is used for selecting significant samples.

#### B/NK/T cell exhaustion single sample GSEA analysis

Is performed in R4.2.0, the markers of B cell exhaustion, NK cell exhaustion, and T cell exhaustion are displayed in Supplementary Table 1.

#### Drug sensitivity prediction

Drug score is calculated by *OncoPredict* package in R4.2.0.

#### Nomogram

Monogram is the visualization of multivariate Cox regression, and it is constructed by the *regplot* package in R4.2.0.

### Statistics

Big data analysis is performed by at least two researchers, independently. All analysis is performed in R4.2.0. *In vitro* experiments, data is performed at least three times independently. *P* < 0.05 is regarded as a statistically significant difference.

### Data availability statement

The raw data can be acquired from corresponding authors.

## RESULTS

### Construction of anoikis-related-gene-based prognosis model in digest system cancers

**Figure 1:**
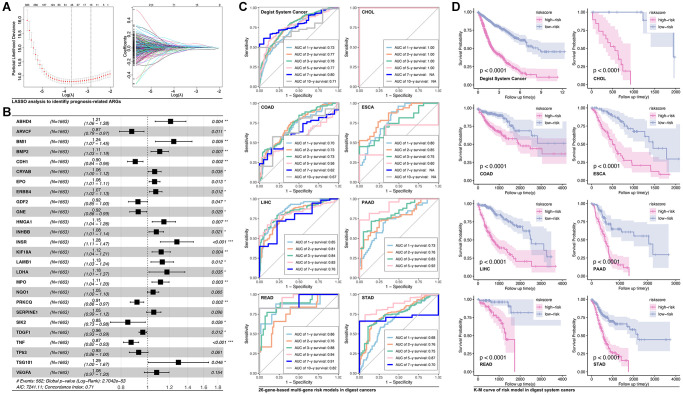
**Anoikis-related genes construct prognosis model in digest system carcinomas.** (**A**) LASSO analysis screens out (**B**) 26 prognosis-related ARGs, amongst which ABHD4, BMI1, BMP2, CRYAB, EPO, ERBB4, HMGA1, INHBB, INSR, KIF18A, LAMB1, LDHA, MPO, NQO1, SERPINE1, TSG101 and VEGFA are risk factors whereas ARVCF, CDH1, GDF2, GNE, PRKCQ, SIK2, TDGF1, TNF and TP53 is protective factors. (**C**) ROC analysis of ARGs-based prognosis model of the whole digest system cancer and single type of digest system cancer, including CHOL, COAD, ESCA, LIHC, ESCA, LIHC, PAAD, READ, STAD. (**D**) K-M analysis of ARGs-based prognosis model.

In order to assess the function of anoikis-related genes (ARGs) in predicting prognosis of digest system cancer, we firstly use LASSO (Figure 1A), by which we filter out 26 prognosis-related ARGs (Figure 1B). These ARGs are put into multivariate cox regression, and ROC analysis is performed to assess the prediction efficiency. For example, AUC value of READ is 0.86 for 1-year survival prediction, 0.76 for 2-year survival prediction, 0.88 for 3-year survival prediction, 0.94 for 5-year survival prediction, 0.91 for 7-year survival prediction and 0.82 for 10-year survival prediction (Figure 1C). Then, ARGs-based multiple gene riskscore is put into Kaplan-Meier analysis, and the results show that higher riskscore group is accompanied with worse prognosis in all types of digest system cancers (Figure 1D).

**Figure 2:**
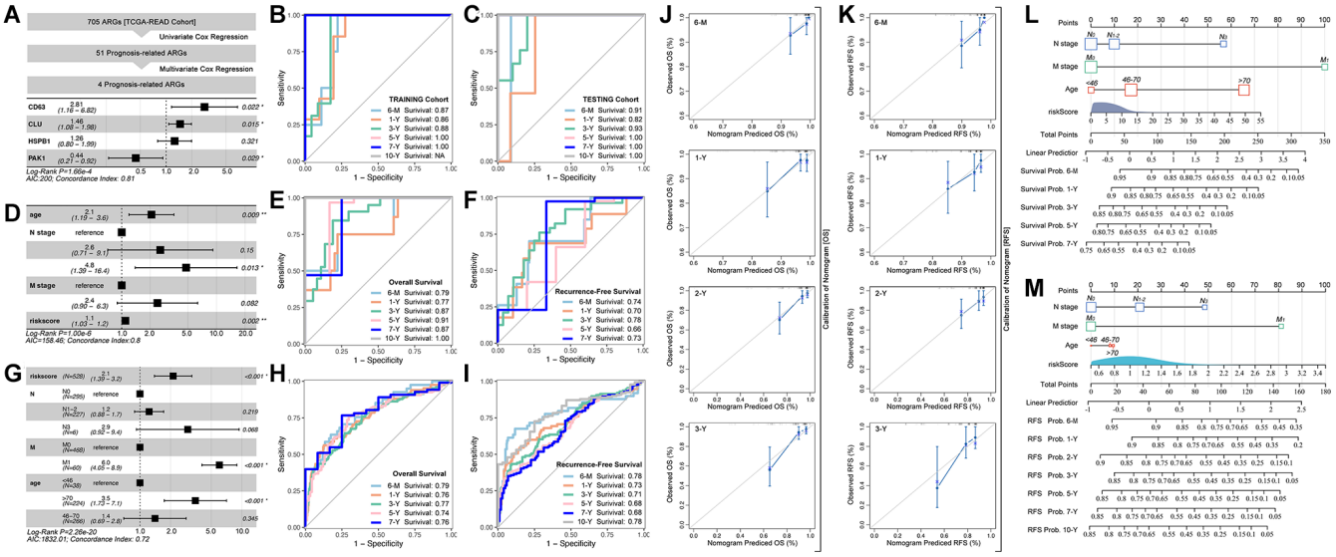
**ARGs-based prognosis prediction model.** (**A**) Multiple gene risk model in TCGA cohort, and its ROC analysis in (**B**) training cohort and (**C**) testing cohort. (**D**) Prognosis prediction model constructed with clinical characteristics and multiple gene riskscore, and its ROC analysis of (**E**) overall survival and (**F**) Recurrence free survival. (**G**) Prognosis prediction model constructed with clinical characteristics and multiple gene riskscore, and its ROC analysis of (**H**) overall survival and (**I**) Recurrence free survival, in GSE39582 cohort. Calibration of prognosis prediction model of (**J**) overall survival and (**K**) Recurrence free survival. Nomogram of (**L**) overall survival and (**M**) Recurrence free survival.

Out of its best performance of ARGs in predicting prognosis in READ, we choose the READ cohort to further analysis. Firstly, 705 ARGs are filtered by univariate cox regression, amongst which 51 are prognosis-related (Figure 2A). Then, multivariate cox regression selects CD63 (HR = 2.81, *p* = 0.022), CLU (HR = 1.46, *p* = 0.015), HSPB1 (HR = 1.26, *p* = 0.321) and PAK1 (HR = 0.44, *p* = 0.029) to construct riskscore (Figure 2A). The AUC value of training cohort is 0.87 for 6-month survival prediction, 0.86 for 1-year survival prediction, 0.88 for 3-year survival prediction, 1.00 for 5-year survival prediction, 1.00 for 7-year survival prediction (Figure 2B), and the AUC value in testing cohort is displayed in Figure 2C. Next, multivariate cox regression selects *Age*, *N-stage*, *M-stag* and *ARG riskscore* to construct final prognosis prediction model (Figure 2D). ROC analysis of overall survival (OS) prediction and recurrence free survival (RFS) are performed, and the results are displayed in Figure 2E. Besides, we also explore the above model in predicting RFS, the results are showed in Figure 2F. To further verify the prediction efficiency of ARG-based riskscore, we apply GEO cohorts (Figure 2G). ROC analysis shows that the AUC value of OS prediction is 0.79 for 6-month survival, 0.76 for 1-year survival, 0.77 for 3-year survival, 0.74 for 5-year survival, 0.76 for 7-year survival (Figure 2H), and the RFS prediction efficiency is also displayed in Figure 2I.

To further describe the prediction efficiency of ARG-based riskscore, calibration analysis is performed for assessing OS prediction (Figure 2J) and RFS prediction (Figure 2K). Besides, the ARG riskscore of OS (Figure 2L) and RFS (Figure 2M) are visualized.

### Non-supervised machine learning to recognize subpopulation of digest system cancer

**Figure 3:**
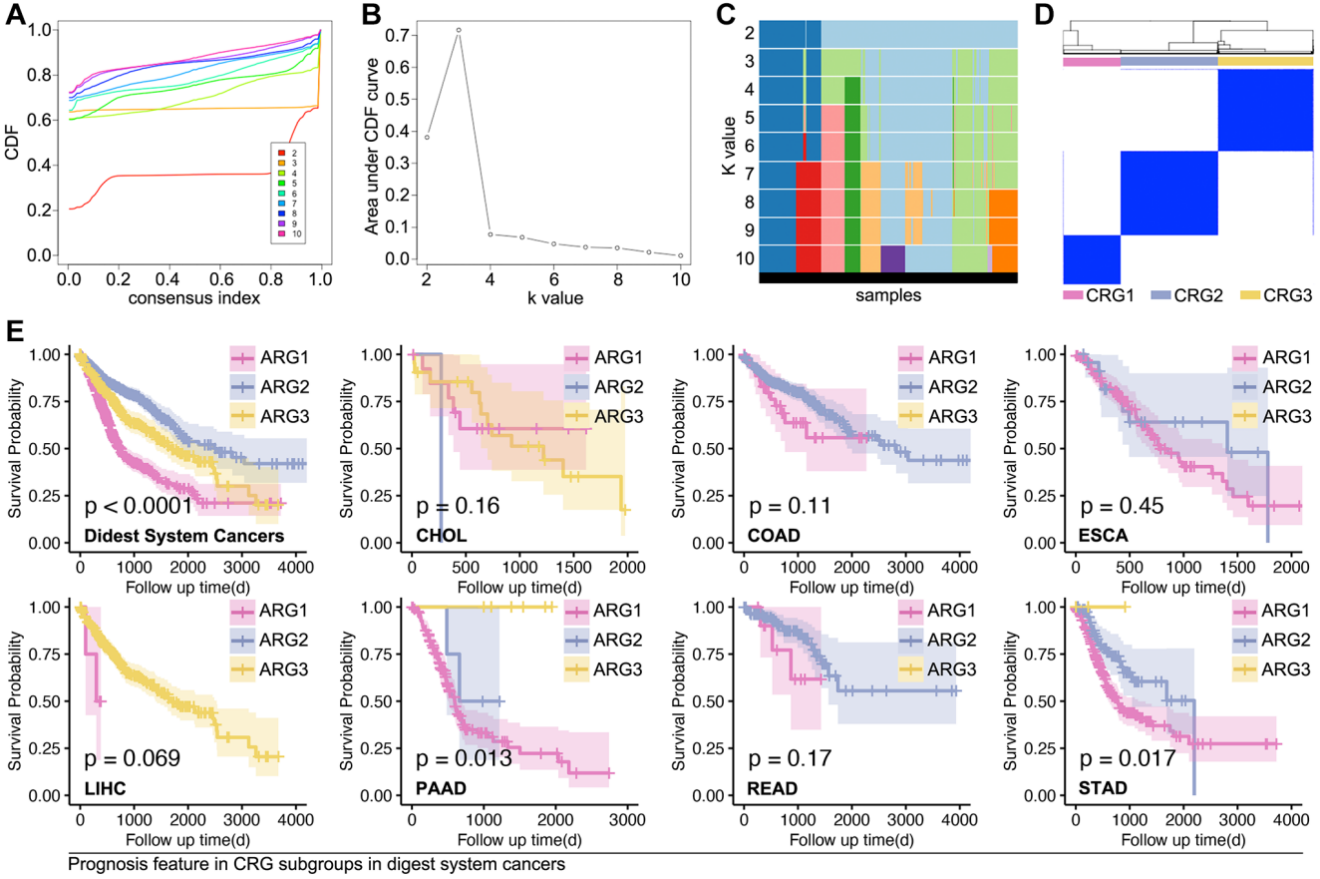
**Supervised machine learning recognizes subpopulations in digest system cancer.** (**A**–**D**) Consensus cluster analysis identifies three ARG subpopulations by *ConsensusClusterPlus* package in R4.2.0 (**E**) K-M analysis explores prognosis differences amongst ARG subpopulations, in which it shows significance in whole cohort of digest system cancers, PAAD cohort and STAD cohort.

Based on the above results, we apply these ARGs to identify subpopulations of digestive system cancer. Consensus cluster analysis gives out grouping suggestions, in which three-grouping is the best strategy (Figure 3A–3D). Then, we explore the prognosis differences amongst ARG subpopulations, and a significant prognosis difference is observed in the whole digest system cohort (*p* < 0.0001, Figure 3E), PAAD cohort (*p* = 0.013, Figure 3E), and STAD cohort (*p* = 0.017, Figure 3E).

### Supervised artificial intelligence constructs ARG recognition model with significant hierarchical prognosis differences

**Figure 4:**
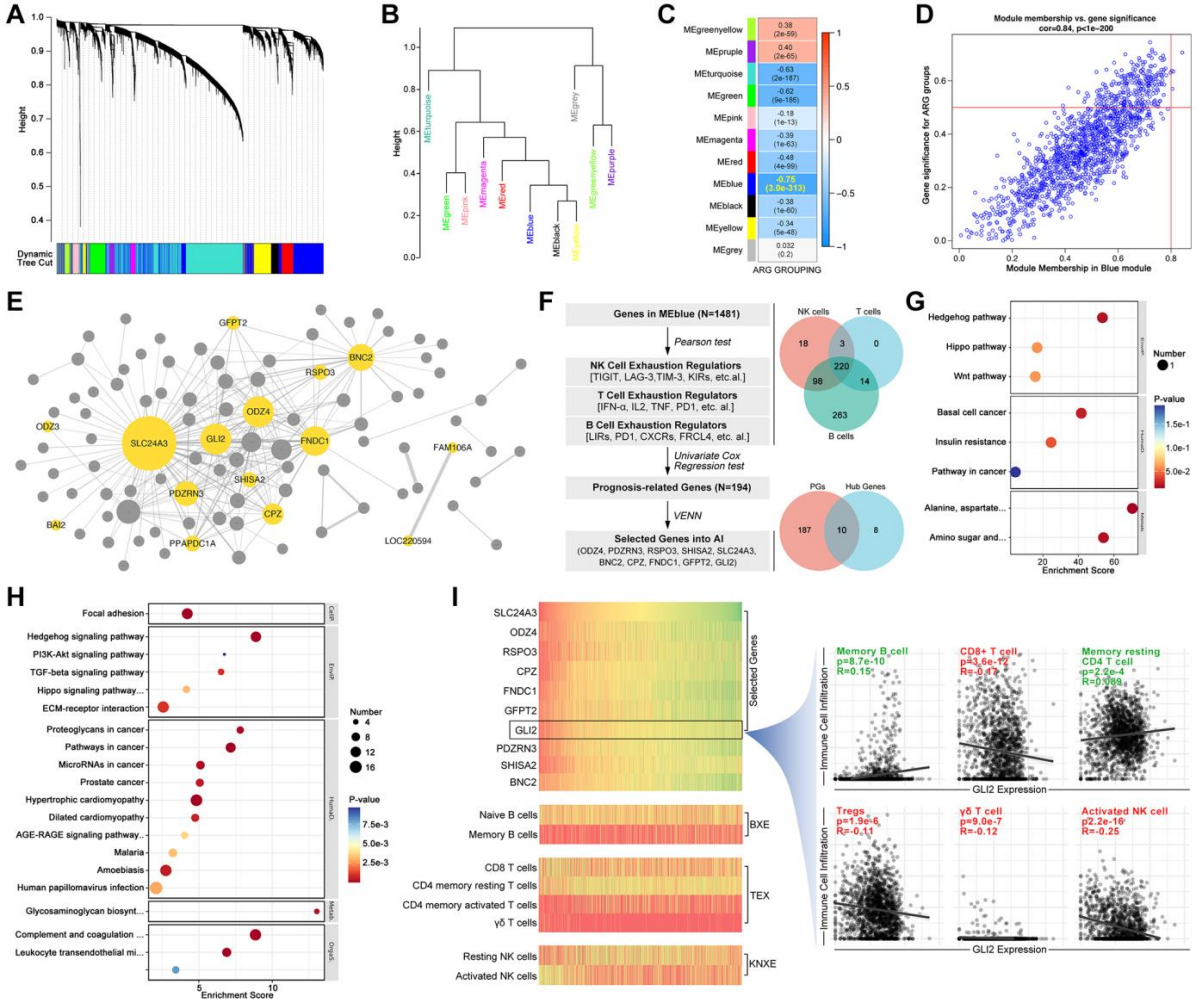
**Screening ARG stratification hubgenes.** WCGNA analysis is performed to collect ARG grouping-related genes, in which (**A**) dynamic tree shows simple sample distribution, and the algorithm finally divides genes into (**B**) 10 groups. (**C**, **D**) Modules show MEblue is closest to ARG grouping (R = −0.75, *p* = 3.0e-313). (**E**) CytoScape constructs network of genes in MEblue, in which ODZ3, BAI2, SLC24A3, PDZRN3, PPAPDC1A, GLI2, SHISA2, CPZ, ODZ4, GFPT2, RSPO3, FNDC1, BNC2, FAM106A and LOC220594 are key genes in ARG grouping. (**F**) Select NK cell exhaustion, B cell exhaustion and T cell exhaustion co-correlated genes in MEblue module, and screened by univariate cox regression, after which 10 genes are selected (ODZ4, PDZRN3, RSPO3, SHISA2, SLC24A3, BNC2, CPZ, FNDC1, GFPT2, GLI2). (**G**) GO pathway analysis shows 10 genes are related to Hedgehog signaling. (**H**) KEGG analysis shows 10 genes are related to Hedgehog signaling. (**I**) Gene expression and immune cell infiltration features in each sample, and GLI2 is selected to display relationship with tumor immunity.

In order to recognize ARG subpopulation in independent cohorts, we use artificial intelligence (AI) to construct a model. Firstly, we use WCGNA analysis to filter hubgenes. As results show, genes are divided into 11 modules within a threshold value of 0.25 (Figure 4A, 4B), and module Blue (MEblue) is recognized as the most important to ARG subgroup identification (R = −0.75, *p* = 3.0e-313, Figure 4C, 4D). Next, CytoScape shows that ODZ3, BAI2, and SLC24A3, et al. are key genes (Figure 4E). Following, genes in MEblue module are put into *pearson test* and *venn* analysis to filter out 220 NK cell exhaustion related, T cell exhaustion related and B cell exhausted relation genes (Figure 4F). After univariate analysis, 10 genes (ODZ4, PDZRN3, RSPO3, SHISA2, SLC24A3, BNC2, CPZ, FNDC1, GFPT2, GLI2) are finally filtered out (Figure 4F). These genes are related to Hedgehog signaling, TGFβ signaling, and immunity processes by KEGG and GO analysis (Figure 4G, 4H). Besides, the expression of these genes is displayed in Figure 4I, likewise the relationship between these genes and immune cell infiltration.

**Figure 5:**
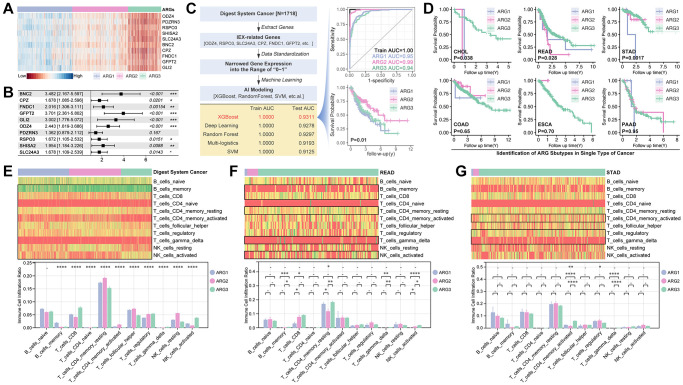
**Machine learning constructs a ARG subpopulation identification model.** (**A**) Expression and (**B**) Hazard ratio of ten Hubgenes in ARG subgroup. (**C**) Five types of machine learning algorithms are performed to construct ARG grouping models based on supervised learning, and XGBoost displays best results that its training AUC is 1.0000 accompanied with testing AUC is 0.9311, and K-M analysis shows difference in prognosis amongst XGBoost identified ARG subpopulations (*p* = 0.01). (**D**) K-M analysis in single type of cancer. Immune cell infiltration in ARG subpopulations in (**E**) whole digest system cohort, (**F**) READ cohort and (**G**) STAD cohort.

The expression profile of the aforesaid 10 genes is displayed in Figure 5A, which shows an obvious significant difference (Figure 5A), and all of these genes are risk factors, except PDZRN3 (Figure 5B). Following, Five AI algorithms are applied to construct models. As results show, XGboost performs best (training AUC is 1.000, and testing AUC is 0.9311) (Figure 5C), and subgrouping shows a significant prognosis difference of prognosis (*p* = 0.01, Figure 5C). As in the single type of digest system cancer, XGboost-identified AGR subgroup shows significant prognosis differences in CHOL (*p* = 0.038), READ (*p* = 0.028) and STAD (*p* = 0.0017) (Figure 5D). Then, immune cell infiltration is explored (Figure 5E–5G). As the Figure 5E–5G show, NK cell infiltration differences are observed in READ cohort (Figure 5E–5G).

**Figure 6:**
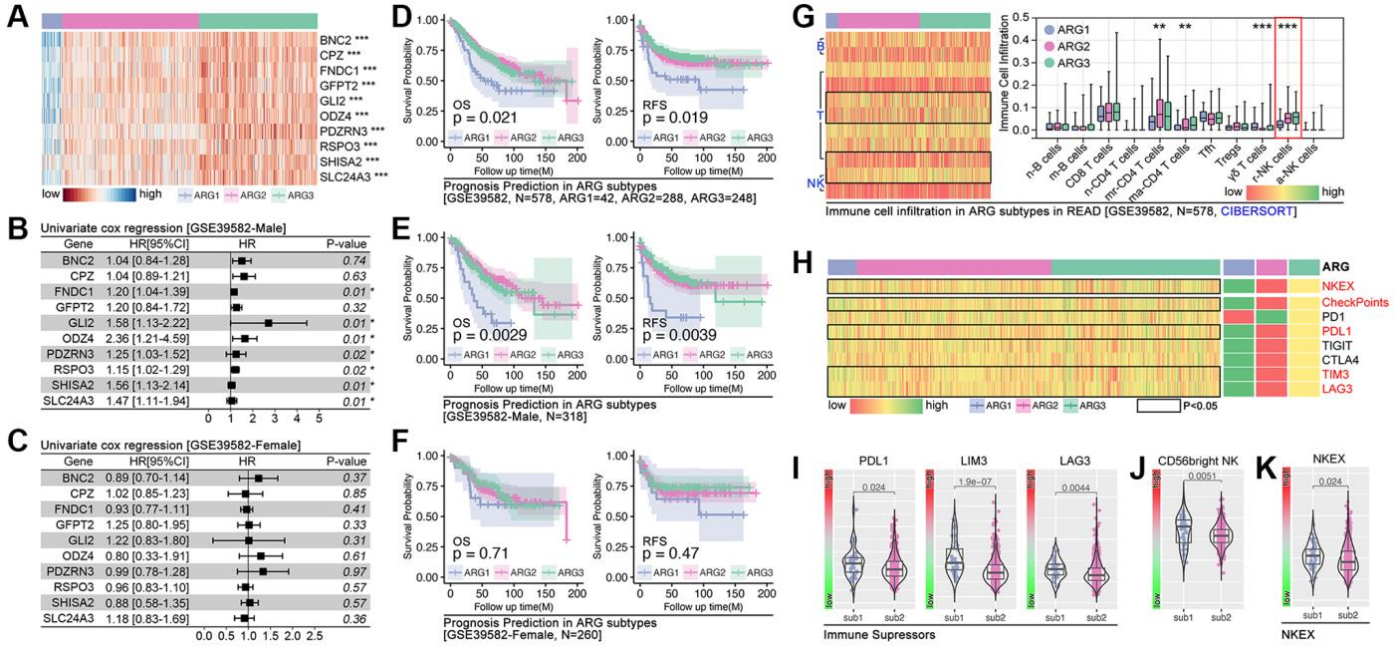
**Independent cohort verification of ARG-based AI model.** (**A**) Gene expression heatmap. (**B**, **C**) Hazard ratio of hubgenes in male cohort and female cohort in GSE39582. (**D**) Prognosis differences in AI model identified ARG subpopulations, in which overall survival (*p* = 0.021) and recurrence free survival (*p* = 00.019) are different in whole cohort, also different in (**E**) male cohort while no significance in (**F**) female cohort. (**G**) Immune cell infiltration in ARG subpopulations in GSE39582 cohort. (**H**–**K**) Immunity checkpoints expression features in ARG subpopulations.

Following, independent cohort (GSE39582) is applied to further verify the above AI model. As the heatmap shows, all of these 10 hubgenes are differently expressed in subpopulations (*p* < 0.001, Figure 6A), and FNDC1 (HR = 1.20, *p* = 0.01), GLI2 (HR = 1.58, *p* = 0.01) and ODZ4 (HR = 2.36, *p* = 0.01), et al. are risk factors in the male cohort (male-GSE39582) (Figure 6B). However, no significant difference is observed in female cohort (Figure 6C). Besides, K-M analysis shows significant difference of prognosis only in cohort of GES39582 (Figure 6D) and male cohort of GSE39582 (Figure 6E), but not in female cohort of GSE39582 (Figure 6F). Besides, NK cell infiltration is significant different in subgroup (Figure 6G). Single- sample GSEA analysis shows significant differences in NK cell exhaustion (NKEX) and checkpoints score (consisting of PD1, PD-L1, TIGIT, TIM3, and LAG-3) amongst subpopulations (Figure 6H). More details are displayed in subpopulation-1 and subpopulation-2 (Figure 6I–6K).

### ARGs-based AI model is an efficient tool to assess drug response in colorectal cancer

**Figure 7:**
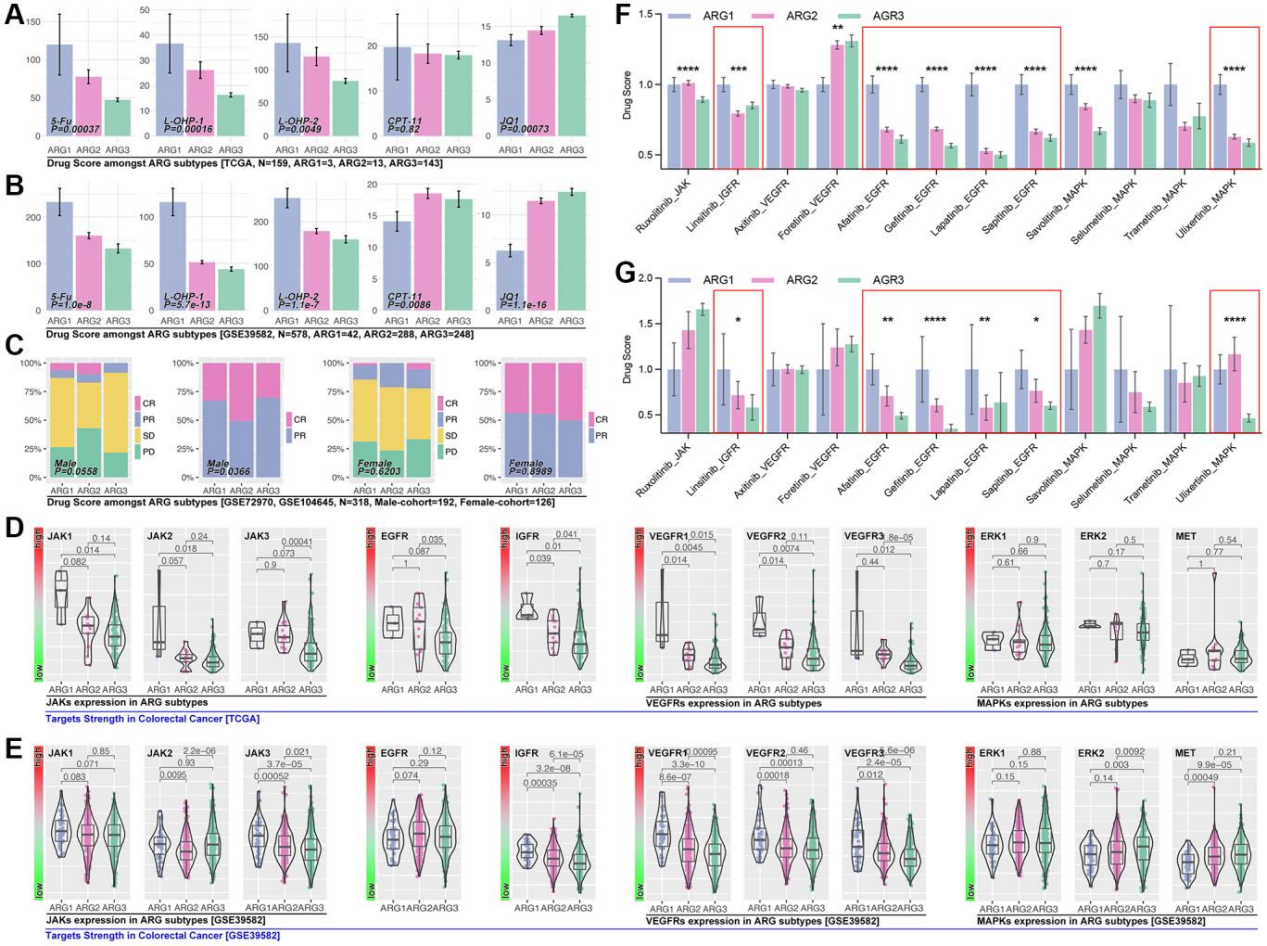
**ARGs-based AI model recognizes drug efficiency.** (**A**) Drug scores of 5-Fu, L-OHP-1, L-OHP-2, CPT-11 and JQ1 in ARG sub groups, calculated by *OncoPredict* package in R4.2.0., data from TCGA, (**B**) GSE39582. (**C**) Clinical trials response in ARG subpopulations identified by AI. Small molecule inhibitor targets expression level in ARG subpopulations in (**D**) TCGA cohort and (**E**) GES39582 cohort. Small molecule inhibitors sensitivity in ARG subpopulations, in which IGFR, EGFR and MAPK inhibitors hold significant differences in (**F**) TCGA cohort and (**G**) GSE39582.

Significant differences in drug score (5-Fu, L-OHP-1, L-OHP-2, JQ1) are observed in ARG subpopulations in the TCGA cohort (Figure 7A). Same results are observed in GSE39582 cohort (Figure 7B). In clinical trials, we only find the significant difference of drug response in the male cohort (*p* = 0.0366, Figure 7C). Next, we explore the popular small molecule targets. As the results display, significant expression differences of JAKs (JAK1, JAK2, JAK3), EGFR, IGFR, VEGFRs (VEGFR1, VEGFR2, VEGFR3) are observed in XGboost-identified ARG subpopulations in TCGA cohort (Figure 7D). In GEO cohort (GSE39582), we observed same results, and ERKs (ERK1, ERK2, ERK3) are expressed differently in ARG subpopulations (Figure 7E). Then we explore those targets’ corresponding inhibitors. Results show that the score of small molecule inhibitors target IGFR (Linsitinib), EGFR (Afatinib, Gefitinib, Lapatinib, Sapitinib), and MAPK (Ulixertinib), is significantly different amongst ARG subpopulations in TCGA cohort (Figure 7F) and GEO cohort (GSE39582, Figure 7G).

### GLI2 is a key ARG in regulating NK cell exhaustion and drug tolerance by non-classical Hedgehog in colorectal cancer

**Figure 8:**
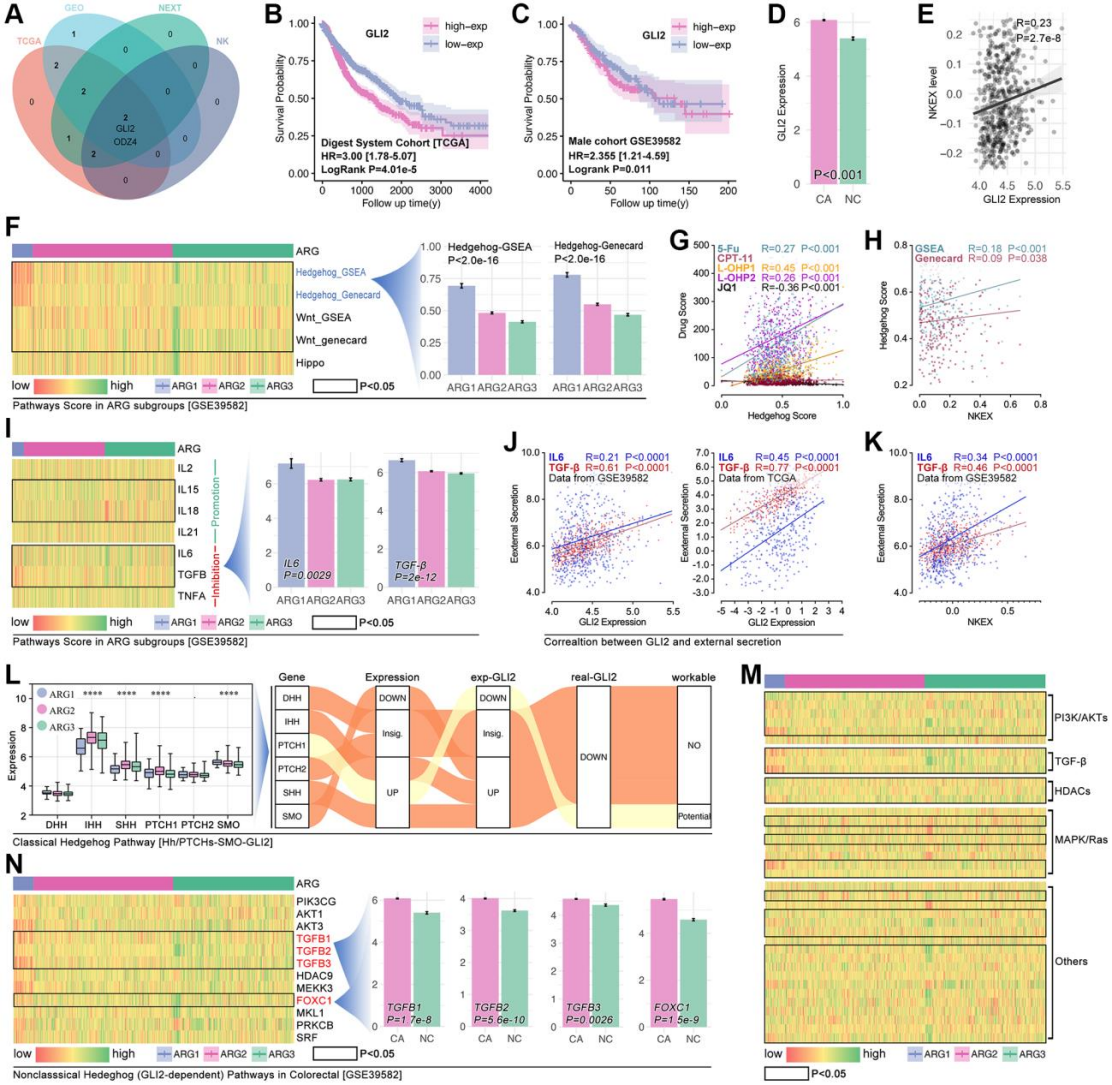
**GLI2 potentially regulates NKE by non-classic Hedgehog signaling to promote drug tolerance and colorectal immunity escape.** (**A**) Multi-cohort association analysis screens out GLI2 and ODZ4 as the closest genes in regulating NKE. K-M analysis for GLI2 in (**B**) TCGA cohort and (**C**) GSE39582 cohort. (**D**) GLI2 expression between colorectal tissues and adjacent tissues. (**E**) Correlation analysis between GLI2 and NKE score. (**F**) Pathways score in ARG subpopulations, and Hedgehog pathway score displays significant differences amongst ARG subpopulations (*p* < 2.0e-16). (**G**) The correlation between Hedgehog pathway score and drug scores. (**H**) Correlation between NKE score and Hedgehog pathway score. (**I**) NKE-related inflammation factors expression in ARG subpopulations. (**J**) Correlation between GLI2 and NKE regulators (IL6, TGFβ). (**K**) Correlation between KNE and NKE regulators (IL6, TGFβ). (**L**) Pathway network between GLI2 and classic Hedgehog pathway. Gene expression characteristics of non-classic Hedgehog signaling in ARG subgroups in (**M**) TCGA cohort and (**N**) GSE39582 cohort.

In order to uncover the mechanisms of ARGs in regulating drug resistance and immunity escape in digest system carcinomas, network analysis is performed. As Figure 8A shows, GLI2 is corelated with prognosis and KEXT (Figure 8A). Results shows that higher expression of GLI2 is related to worse prognosis in READ, both in the TCGA cohort and GEO cohort (Figure 8B, 8C), and the expression level of GLI2 is higher in colorectal cancer tissues as compared with normal tissues (*p* < 0.001, Figure 8D). Pearson test shows GLI2 is positively correlated with NKEX (r = 0.23, *p* = 2.7e-8). According to the KEEG and Go analysis results in Figure 4, we explore the differences of this pathway in XGboost-identified ARG subpopulations, and the results show that Hedgehog signaling score is different amongst ARG subgroups (*p* < 2.0e-16, Figure 8F). As previous studies report, GLI2 is closely related to Hedgehog in regulating tumor progression [12, 13], so GLI2 is put into further analysis. We explore the correlation between Hedgehog signaling score and drug score, and the results show that Hedgehog signaling score is positively correlated with 5-Fu (r = 0.27, *p* < 0.001), L-OHP-1 (r = 0.45, *p* < 0.001), L-OHP-2 (r = 0.26, *p* < 0.001) (Figure 8G). Besides, Hedgehog signaling score is positively correlated with NKE (Figure 8H). Those results imply that GLI2 probably regulates Hedgehog signaling to interfere NK cell immunity and drug resistance in colorectal cancer.

After a literature review, we collect factors that directly regulate the function of NK cells, and the gene list is IL2, IL15, IL18, IL21, all of which promote NK cell-mediated tumor death, while IL6, TGFβ and TNFα promote NK cell exhaustion [14–16]. In the heatmap, we find different expressions of IL15, IL18, IL6 and TGFβ in XGboost-identified ARG subpopulations in the GEO cohort (Figure 8I). *Pearson test* analysis shows that GLI2 expression is closely correlated with IL6 (r = 0.45, *p* < 0.0001) and TGFβ (r = 0.77, *p* < 0.0001) in TCGA cohort, and same results are observed in GEO cohort (Figure 8J). Besides, there is significant correlation between NKEX and IL6 (r = 0.34, *p* < 0.0001) or TGFβ (r = 0.46, *p* < 0.0001) (Figure 8K).

As Figure 8L shows, the expected Hedgehog-mediated GLI2 change isn’t consistent with the real change of GLI2 (Figure 8L). This implies classic Hedgehog signaling is not key in GLI2-induced drug resistance and NEXT. For non-classic Hedgehog signaling, we enroll PI3K/AKT, TGFβ signaling, HDACs, MAPK/Ras, and other reported targets which are involved in Hedgehog signaling in cancer. In TCGA cohorts, we observe that almost all of the above genes are differently expressed in XGboost-identified ARG subpopulations (Figure 8M), while only TGFβ (TGFβ1-3) and FOXC1 are different expression in ARG subpopulations in GEO cohort (Figure 8N).

### Down-regulation of GLI2 decreases drug tolerance in colorectal cancer and gastric cancer

**Figure 9:**
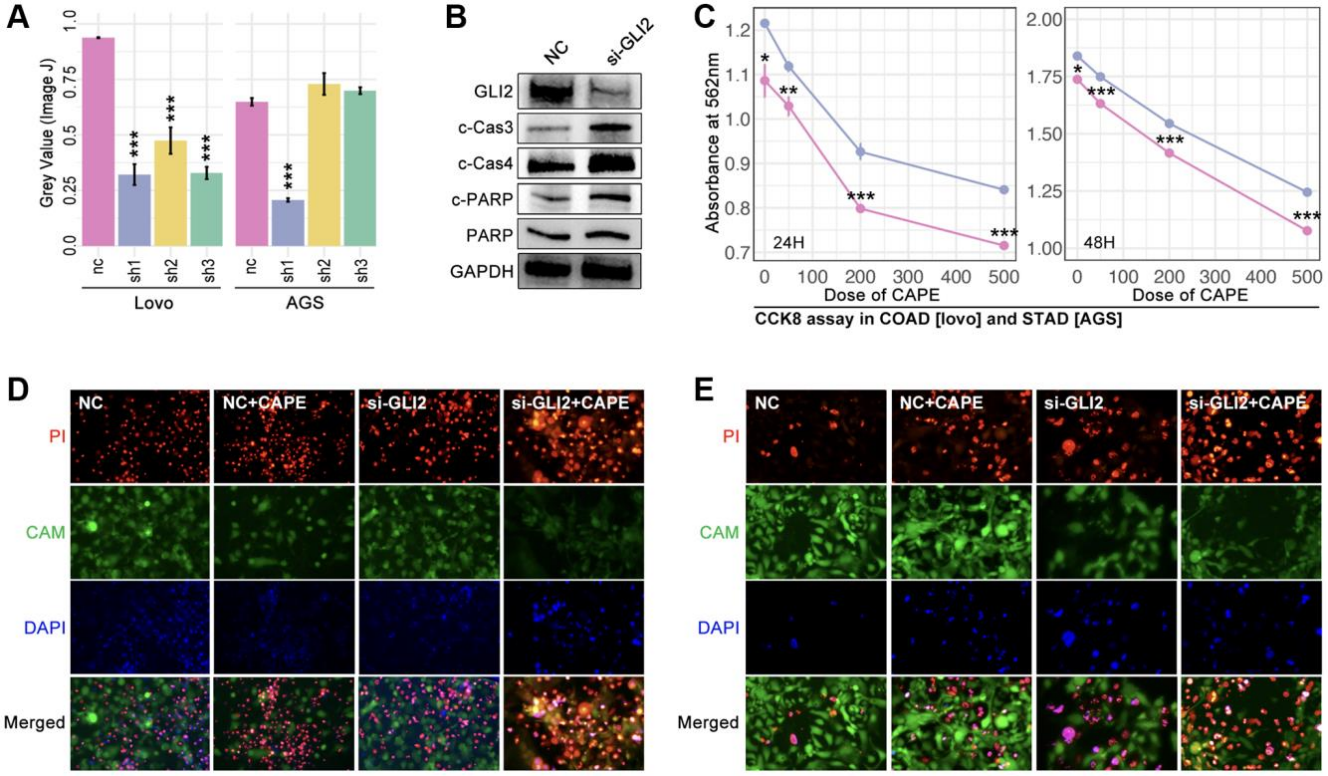
**GLI2 promoted drug tolerance.** (**A**) Expression level of GLI2 after siRNA treatment in colorectal cancer cell (lovo) and gastric cancer by (a) PCR assay and (**B**) WB. (**C**) CCK-8 assay. Alive and dead cell staining in (**D**) colorectal cancer and (**E**) gastric cancer.

Small interfere RNA (siRNA) technology decreases the GLI2 expression in lovo cells and AGS cells (Figure 9A, 9B). And the down-regulation of GLI2 significantly decreases the cell viability as being treated with CAPE in different concentration (Figure 9C). Then, alive and dead assay is performed, and results show that down-regulation of GLI2 enhances the cell toxicity of CAPE in colorectal and gastric cancer cell lines (Figure 9D, 9E).

### GLI2-TGF-beta axis promotes NKEX by non-classical Hedgehog pathway in READ

**Figure 10:**
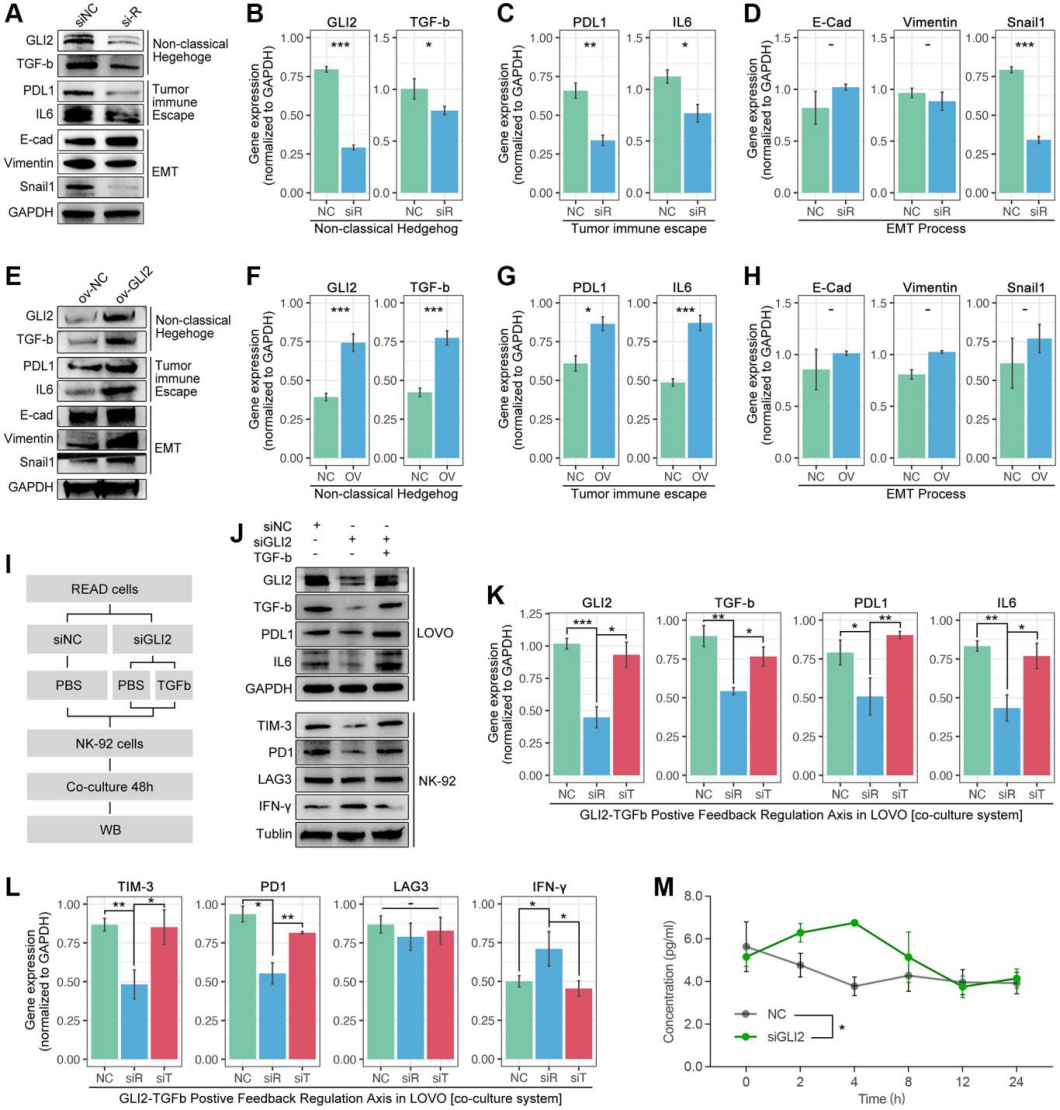
**GLI2 promotes NEXT by regulating TGF-beta-mediated non-classical Hedgehog signaling.** (**A**–**D**) Down-regulation of GLI2 is accompanied by down-regulation of PD-L1, TGF-beta, IL6 and snail1. (**E**–**H**) Up-regulation of GLI2 is accompanied with increased expression of PD-L1, TGF-beta, and IL6. (**I**) Co-culture process. (**J**–**L**) Down-regulation of tumor-derived GLI2 decreases tumor-derived TGF-beta, PDL1 and IL6, likewise the NK-derived TIM-3 and PD1, while NK-derived IFN-gamma is increased. TGF-beta active protein powder restores the expression of tumor-derived PDL1 and IL6, and NK-derived TIM-3 and PD1. (**M**) Down-regulation of tumor-derived GIL2 leads increased secretion of NK-derived IFN-gamma, detected by Elisa.

Down-regulation of GLI2 in LOVO cells decreases the expression level of TGF-beta, PDL1, IL6 and snail1, while the expression level of E-cadherin and vimentin are not interfered (Figure 10A–10D). Then, we apply recombinant plasmid to increase the expression of GLI2, and results show that TGF-beta, PDL1 and IL6 are obviously increased, while no significant change is observed in E-cadherin, vimentin and snail1 (Figure 10E–10H). To further uncover the GLI2-TGF-beta pathway in NEXT, we performed co-culture (Figure 10I). As Figure 10J shows, down-regulation of tumor-derived GLI2 leads down-regulation of PDL1, TGF-beta and IL6 in lovo cells, and it also leads down-regulation of TIM-3, PD1 and IFN-gamma in NK cell line (NK-92) (Figure 10J–10L). Then, we apply TGF-beta active protein powder. As the results displayed in Figure 10, TGF-beta protein restores the decreased expression of tumor-derived PDL1 and IL6 and NK-derived TIM-3 and PD1 (Figure 10J–10L). Collectively, we find down-regulation of tumor-derived GLI2 leads up-regulation of NK-derived IFN-gamma (co-culture) (Figure 10M).

## DISCUSSION

Due to the delayed diagnosis, colorectal cancer is developing as a formidable disease in tumor treatment, which means more than 80% of patients with colorectal cancer are in the advanced stage at first diagnosis [2]. Although microsatellite instability (MSI) detection is useful for the determination of making drug therapy strategy in CRC, partial patients with a high score of MSI still response to immunotherapy weakly [17], and low score of MSI with a weak response to 5-fu-based chemotherapy [18]. Therefore, it’s necessary to construct a new drug sensitivity assessment system as additional evidence for making individual therapy regimens in colorectal cancer.

Multi-gene-based models are reported in various kinds of tumors, such as liquid-liquid related gene-based risk model in breast cancer [19]. Besides, ferroptosis-derived, immunogenic cell death-derived, autophagy-derived, and cuproptosis-derived genes are also applied in making a tool to assess prognosis and drug response [20–22]. Recently, Anoikis-related genes are reported to be used in assessing prognosis and immune cell infiltration in liver cancer, lung cancer, and also in colorectal cancer [10, 23]. To our knowledge, overcoming anoikis is a necessity during tumor metastasis and invasion. In fact, developing evidence implies that anoikis is also involved in drug resistance in tumors, and also related to immunotherapy [24]. In this study, ARGs are applied to construct a prognosis model, and it shows a relative more efficient ability of prognosis prediction in CHOL, READ, and LIHC (Figure 1C). In further exploration, independent cohort of READ (GEO data) is applied, and independent verification shows ARGs hold potential in predicting clinical outcomes, such as OS and RFS (Figure 2J–2M). Those results imply that the ARG-based risk model can be considered as the adjuvant tool in clinical outcome assessment.

AI is already applied in medicine, such as imaging-assisted interpretation. Nowadays, more and more studies show that AI is powerful in identifying or redefining tumor subtypes. For an illustration, the AI-based immunotherapy response assessment system recognizes the responsiveness of anti-PD-L1 treatment in melanoma, kidney cancer, and thyroid cancer [25–27]. To the importance of the immune cell exhaustion in immunotherapy resistance, WCGNA is used to find hubgenes that play key roles in ARG subpopulations identification and immunity escape (Figure 4F). And following, five types of supervised AI algorithms are applied to construct models, all of which have pretty efficient in recognizing ARG subgroups, especially for XGboost (training AUC is 1.000 and testing AUC is 0.9311, ARG1-AUC is 0.95, ARG2-AUC is 0.99, and ARG3-AUC is 0.94. Figure 5C). In external cohort verification, constructed AI model recognizes ARG subpopulations and also distinguishes prognosis differences in colorectal cancer (Figure 6A, 6D). Interestingly, there are no significant prognosis difference amongst AI-recognized ARG subgroups in the female cohort (Figure 6F). To our knowledge, the tumoral biology of CRC is significantly different between female and males [28]. For example, significant differences in progression-free survival (PFS) are observed in the male cohort with combining treatment of capecitabine and bevacizumab, while it’s no significant difference in the female cohort [28]. In fact, a higher incidence and death rate of CRC is observed in male cohorts as compared to females [1].

To further explore the role of AI in assessing drug response, the *OncoPredict* algorithm is applied. And the results show a significant hierarchical stratification of drug score in the CRC cohort, both in the TCGA cohort and GEO cohort (Figure 7A, 7B). And, that is also verified in clinical trials in the male cohort (Figure 7C). Besides, the response of small molecule inhibitors (SMI) in CRC is also explored. Excitingly, the expression level of IGFR, EGFR, and MAPK show significant differences amongst ARG subtypes, accompanied by significant differences in SMI score (Figure 7F, 7G). Those foregoing results imply that ARG combining AI exhibits powerful potential ability in drug responsivity prediction and prognosis prediction.

In order to uncover the mechanisms of ARG in regulating drug resistance and immune cell exhaustion, *venn* analysis is performed to screen out GLI2 as a candidate in determining immune cell destiny in CRC (Figure 8A–8E). In fact, GLI2 is reported to promote chemotherapy resistance via regulating HIF-1α and TGF-β2 in CRC [29], and it also enhances Hedgehog signaling to lead to GLI2-dependent drug tolerance in CRC [30]. In this study, the Hedgehog signaling score is highest in the ARG1 subgroup, in which group it also holds the highest drug score (Figures 7A, 7B, and 8F). Meantime, the correlation between the Hedgehog signaling score and NEXT is positive (Figure 8H). That result implies that Hedgehog signaling is close to drug resistance in ARG-mediated drug tolerance. In further analysis, the direct regulators of NK cell viability and ability are collected by a literature review (Figure 8I) [14]. And it is explicit that viability inhibitors (IL6, TGFβ) of NK cells are higher expression in the ARG1 subpopulation, and the correlation between those regulators and NKEX is positive (Figure 8K). In addition, the correlation between GLI2 and direct regulators of NK cells is also positive (Figure 8J). Based on the above evidence, GLI2 probably regulates NEXT through non-classical Hedgehog signaling in CRC, and this hypothesis is verified by *in vitro* experiments. As the results show, down-regulation of GLI2 decreases the expression of TGF-beta, while the up-regulated TGF-beta restores the expression of siRNA-mediated down-regulation of GLI2. This implies that a cooperative expression model exists in GLI2 and TGF-beta. Besides, we find down-regulation of tumor-derived GLI2 decreases the expression of tumor-derived PDL1 and IL6, and it also leads down-regulation of NK cell-derived PD1 and TIM-3, all of which are restored by adding TGF-beta active protein (Figure 10). These aforesaid results imply that TGF-beta-mediated non-classical Hedgehog pathway is pivotal in GLI2-mediated NEXT.

## CONCLUSION

In this research, we apply AI to construct a prognosis prediction model and drug response prediction tools. We screen out GLI2 is a key ARG that promotes drug tolerance and tumor immunity escape via the TGF-beta/non-classical Hedgehog signaling pathway. However, this study doesn’t uncover the mechanisms of how tumor-derived GLI2/TGF-beta axis regulates NK cell activity.

## Supplementary Materials

Supplementary Table 1
