# Supplementary material for: Anoikis regulator GLI2 promotes NC cell immunity escape by TGF-β-mediated non-classic hedgehog signaling in colorectal cancer: based on artificial intelligence and big data analysis
Source: Aging (Albany NY). 2023 Dec 29;15(24):14733–48. doi: 10.18632/aging.205283 (PMC10781491; doi:10.18632/aging.205283)
Supplement: Supplementary Table 1 [file aging-15-205283-s001.pdf]

SUPPLEMENTARY TABLE

Supplementary Table 1. The markers of immune cell.

TEX	BEX	NEX
TIGIT	PDCD1	TIGIT
LAG3	FCRL4	LAG3
HAVCR2	SIGLEC6	HAVCR2
PDCD1	LAIR1	PDCD1
BTLA	FCGR2B	KLRC1
CD160	CD38	FCGR3A
CD244	CD70	CD96
CTLA4	CD72	KLRK1
IFNA1	CD86	NCR3
INFB	FAS	NCR2
IL2	ITGAX	NCR1
GZMA	CXCR3	DNAM1
TNF	CXCR4	FCGR3B
PRF1	CXCR5	ITGA2
GZMB	TNFRSF13C	LAMP1
IFNA1	CCR6	IFNG
IFNA2	CCR7	TNF
IFNA4	CR2	PRF1
IFNA5	CD22	GZMA
IFNA6	PMP22	KIR2DL1
IFNA7	SELL	KIR2DL2
IFNA8	LILR1	KIR2DL3
IFNA10	LILR3	KIR2DL4
IFNA11P	LILR4	KIR2DP1
IFNA12P	LILR5	KIR2DS4
IFNA13	LILR6	KIR2DS6
IFNA14	LILRB1	KIR3DL0
IFNA16	LILRB2	KIR3DL1
IFNA17	LILRB3	KIR3DL2
	LILRB4	KIR3DL3
	LILRB5	KIR3DL7
	LILRB6	KIR3DP1
	LILRB7	KIR3DX1
		KIR3DS2P
		GZMB
